# MultiFuzz: A Coverage-Based Multiparty-Protocol Fuzzer for IoT Publish/Subscribe Protocols

**DOI:** 10.3390/s20185194

**Published:** 2020-09-11

**Authors:** Yingpei Zeng, Mingmin Lin, Shanqing Guo, Yanzhao Shen, Tingting Cui, Ting Wu, Qiuhua Zheng, Qiuhua Wang

**Affiliations:** 1 School of Cyberspace, Hangzhou Dianzi University, Hangzhou 310000, China; yzeng@hdu.edu.cn (Y.Z.); lin_mingmin@163.com (M.L.); yanzhaoshen@hdu.edu.cn (Y.S.); cuitingting@hdu.edu.cn (T.C.); zheng_qiuhua@163.com (Q.Z.); wangqiuhua@hdu.edu.cn (Q.W.); 2 State Key Laboratory for Novel Software Technology, Nanjing University, Nanjing 210000, China; 3 School of Cyber Science and Technology, Shandong University, Jinan 250000, China; guoshanqing@sdu.edu.cn; 4 Science and Technology on Communication Security Laboratory, Chengdu 610041, China; 5 Hangzhou Innovation Institute, Beihang University, Hangzhou 310000, China

**Keywords:** coverage-based fuzzing, network protocol, publish/subscribe, multiparty-protocol fuzzer, MQTT, CoAP, IoT, Preeny, security, desock

## Abstract

The publish/subscribe model has gained prominence in the Internet of things (IoT) network, and both Message Queue Telemetry Transport (MQTT) and Constrained Application Protocol (CoAP) support it. However, existing coverage-based fuzzers may miss some paths when fuzzing such publish/subscribe protocols, because they implicitly assume that there are only two parties in a protocol, which is not true now since there are three parties, i.e., the publisher, the subscriber and the broker. In this paper, we propose MultiFuzz, a new coverage-based multiparty-protocol fuzzer. First, it embeds multiple-connection information in a single input. Second, it uses a message mutation algorithm to stimulate protocol state transitions, without the need of protocol specifications. Third, it uses a new desockmulti module to feed the network messages into the program under test. desockmulti is similar to desock (Preeny), a tool widely used by the community, but it is specially designed for fuzzing and is 10x faster. We implement MultiFuzz based on AFL, and use it to fuzz two popular projects Eclipse Mosquitto and libCoAP. We reported discovered problems to the projects. In addition, we compare MultiFuzz with AFL and two state-of-the-art fuzzers, MOPT and AFLNET, and find it discovering more paths and crashes.

## 1. Introduction

Fuzzing [1,2] is an important way to discover vulnerabilities in programs. The basic idea of fuzzing is to feed different inputs into a program under test (PUT) and keep monitoring its status for any misbehavior. Coverage-based fuzzing [3,4,5] is categorised as greybox fuzzing [2,6]. Different from traditional blackbox fuzzing [7,8], coverage-based fuzzing monitors the internal execution paths of inputs, and saves the inputs as further mutation seeds if they exercise any new and interesting paths. Though coverage-based fuzzing usually does not need sophisticated program analysis or the grammar of program input like whitebox fuzzing [9], it is shown to be able to gradually exercise different parts of the program and discover many vulnerabilities [3]. Now, coverage-based fuzzing is being both used by the security industry [3,10] and researched by the academia [5,11,12,13,14,15,16].

Different network protocols are proposed in the Internet of things (IoT) domain [17,18,19,20] to fit the unique requirements of IoT, and several important protocols support the publish/subscribe model [19]. For example, the Message Queue Telemetry Transport (MQTT) protocol [21] uses the publish/subscribe model as its core design, and the Constrained Application Protocol (CoAP) [22] supports to “observe” resources (a similar publish/subscribe model) by using a protocol extension [23]. The publish/subscribe model provides loose coupling and scalability to the IoT network [19]: (i) publishers and subscribers do not need to know the existence of each other, and do not need to be online at the same time, (ii) one publisher could publish data to many subscribers and one subscriber could subscribe data from many publishers, i.e., supporting a many-to-many communication model. In this paper we focus on the fuzzing of two publish/subscribe protocols, MQTT and CoAP. They are widely used in the IoT network, and are also supported by IoT cloud providers like Amazon and Microsoft [24,25].

Currently, there are mainly two methods for network protocol fuzzing. The first method belongs to blackbox fuzzing [19]. It includes general fuzzing tools like Boofuzz (Sulley) [26] and Peach [8], and tools designed for specific protocols like TLS-Attacker [27]. These tools usually need users to write scripts or codes to describe the formats of network messages and the transitions of protocol states. They require the expertise on the protocols to get good fuzzing results, and the scripts and codes need to be updated accordingly when the protocols have new versions. The second method belongs to greybox fuzzing, which is to adapt coverage-based fuzzing tools to the fuzzing of network protocols. The method is more promising since it generally does not need to know protocol specifications or write codes. However, it needs a way to feed fuzzing inputs into the network program under test. One way is to use desock (a module of Preeny) [28] to hook the socket functions like socket() and accept() (recommended by AFL [3] for no code modifications required). The hooking is usually done by LD_PRELOAD, and the sockets returned to the PUT are hijacked to send (or receive) data to (or from) stdout (or stdin). We further explain the design of desock later in Section 4.4. Another way is to send inputs through ordinary sockets like AFLNET [15]. The last way is to modify the program source codes to make the program read packets directly from memory buffers other than real network interfaces, like fuzzing openssl in the Google OSS-Fuzz project [10]. The first two ways may introduce performance bottleneck (as we later shown in Section 5.4). The last way may be not trivial since the modification may need code refactoring.

**Figure 1:**
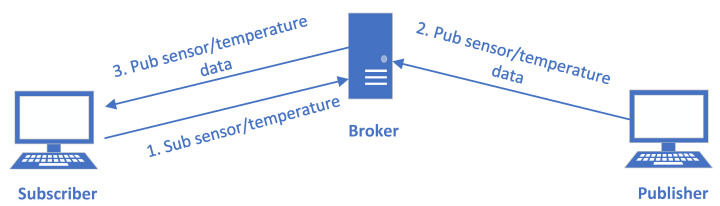
A typical publish/subscribe process of the Message Queue Telemetry Transport (MQTT) protocol (some CONNECT and ACK messages are omitted for simplicity).

We can see that coverage-based fuzzers need less preparation before fuzzing, however, there is another special problem when using them to fuzz the publish/subscribe protocols in IoT. Existing coverage-based fuzzers implicitly assume that there are only two parties in the network protocols (the fuzzers pretend to be one party when fuzzing another party), but there are three parties in the publish/subscribe protocols. Considering a typical process of the MQTT protocol [21,29] in Figure 1, a subscriber subscribes to the sensor/temperature topic first. When a publisher publishes a value to the topic, the subscriber will receive the published value later. Such a process cannot be simulated by existing coverage-based fuzzers, this is because only the messages of a single connection are included in a fuzzing input, and the fuzzer initiates only a single connection for each input [3,10,15]. Thus, even if the fuzzer successfully simulates as a subscriber and sends the first SUBSCRIBE message in the process, it cannot receive the third PUBLISH message, because there is no publisher sending the second PUBLISH message during a fuzzing execution (also the broker server under fuzzing is restarted for each input). The fuzzer may receive the PUBLISH message if it happens to subscribe to some built-in topics, but it cannot make the broker server run the whole dynamic publish/subscribe process shown in Figure 1. If there is a vulnerability in the corresponding execution path in the broker server, the fuzzer cannot discover it. So in general, existing coverage-based fuzzers are not sound for such multiparty protocols.

In this paper, we propose a coverage-based multiparty-protocol fuzzer called MultiFuzz. We compare MultiFuzz with existing fuzzers in Table 1. MultiFuzz does not need any protocol specifications, or any coding by the users. It can initiate multiple connections to a PUT, which enables it to fuzz the IoT publish/subscribe protocols like MQTT and CoAP (Please note that existing greybox fuzzers like AFL [3] and AFLNET [15] could still be used to fuzz the publish/subscribe protocols, i.e., initiating a single connection to the PUT to simulate one party in the protocols, although they may intrinsically miss some paths as we illustrated previously. Blackbox fuzzers like Boofuzz (Sulley) [26] do not restart the PUT after each fuzzing input; therefore, they naturally support multiparty protocols since they may simulate the multiple connections to the PUT by using multiple fuzzing inputs). MultiFuzz has a new module desockmulti to feed network messages into the PUT, and is 10x faster than desock (Preeny). In order to stimulate the state transitions of network protocols, MultiFuzz uses a message mutation algorithm to mutate inputs at a higher level first. MultiFuzz is coverage-based hence it belongs to the greybox category.

Specifically, our paper makes the following contributions:

- We propose a multiparty-protocol fuzzer, MultiFuzz, to soundly support the fuzzing of publish/subscribe protocols. The fuzzer could initiate multiple connections to a PUT, and has a new seed format for storing all messages of the connections in a single seed input.
- We propose a message mutation algorithm to mutate message sequences in a seed input, to efficiently stimulate the state transitions of protocols. The mutation algorithm considers the multiple connections stored in a seed as well.
- We design and implement desockmulti for feeding network messages to a PUT. Previously the community usually uses the desock module of Preeny together with AFL to fuzz network services, but desock supports one connection only. We use a new design to support more than one connection, and further optimize desockmulti to be 10x faster than the widely used desock tool (Section 5.4) (We plan to opensource desockmulti after the publication of this paper).
- We implement MultiFuzz based on AFL, and use MultiFuzz to fuzz two popular projects, Eclipse Mosquitto (an MQTT broker) [29], and libCoAP (a CoAP library) [30]. We reported our found vulnerabilities to the projects and were acknowledged (Section 5.5). We also show that MultiFuzz outperforms AFL, and state-of-the-art fuzzers MOPT [14] and AFLNET [15] in finding program paths and crashes (e.g., finding program paths 44.6% more than AFLNET, 126.6% more than AFL, and 125.4% more than MOPT, when fuzzing Eclipse Mosquitto).

The rest of the paper is organized as follows. In Section 2 we review related work. Then in Section 3 we give a brief introduction to MQTT and CoAP. In Section 4 we describe MultiFuzz in detail. We give experiment results in Section 5, and conclude the paper in Section 6.

## 2. Related Work

Fuzzing [1,2,6,31] now is a widely used technique to discover vulnerabilities in programs. The basic idea of fuzzing is to feed different and even abnormal inputs into a PUT, and keep monitoring its status to check if the program is crashed or misbehaving [1]. Fuzzers could be classified into three categories: blackbox, greybox and whitebox [2,6]. Blackbox fuzzers [7,8] could only monitor the input/output of a PUT, and some of them like Peach may know the structure of inputs [8]. Most traditional fuzzers are in this category [2]. Whitebox fuzzers [9,32] use much more internal information of a PUT, e.g., through symbolic execution. Greybox fuzzers take the middle way that they collect some internal information from a PUT. For example, coverage-based fuzzers collect the coverage information of inputs [2,6]. Greybox fuzzers usually run faster than whitebox fuzzers and utilize more information than blackbox fuzzers [6], and are good choices if we want higher coverage and discovering “hidden” bugs [6]. Modern fuzzers like AFL [3], libFuzzer [4] belong to this category. Greybox fuzzing (mainly coverage-based) has already been widely used in the security industry [3,10]. It is also a hot research topic; many fuzzers like AFLFast [5], CollAFL [11], Angora [12], QSYM [13], MOPT [14], and IJON [16] are proposed.

Fuzzing network protocols (i.e., network services/programs) is known to be difficult [15]. This is because the input is a sequence of messages, but not a single file as in traditional fuzzing, and it needs a way to feed the input into a PUT. Existing network protocol fuzzing approaches could be divided into two categories:

- **Blackbox network-protocol fuzzing** [19]. It includes general protocol fuzzing tools like SPIKE [33], PROTOS [34], SNOOZE [35], LZFuzz [36], Boofuzz (Sulley) [26], and Peach [8], as well as tools specially designed for some protocols, like TLS-Attacker [27] for the Transport Layer Security (TLS) protocol, and MTF [37] for the Modbus protocol. Most of these tools need users to tell the formats of network messages, and some of them also support users to provide the transition rules of protocol states [8]. For the general protocol fuzzing tools, users need to provide such information by scripts [26,33,34] or xml files [8,35]. For the tools specially designed for some protocols, such information is provided by tool developers. Most of these tools pretend as clients to feed inputs into the network programs, and some tools like LZFuzz [36] act as man-in-the-middle (MITM) proxies to modify messages between the client and the server. Blackbox fuzzing usually requires to write scripts, xml files, or codes following the protocol specifications, and needs to update them accordingly when the protocols have new versions. In addition, blackbox fuzzing may be more suitable for discovering “shallow” bugs, comparing with greybox and whitebox fuzzing [6].
- **Greybox network-protocol fuzzing** [3,10,15]. The general coverage-based fuzzing tools like AFL [3] and libFuzzer [4] are used to fuzz network protocols as well. Usually users do not need to know protocol specifications or write any scripts/codes, instead, they prepare (e.g., by recording) some messages as seed inputs. However, since tools like AFL [3] were used to fuzz programs using files/stdin/memory buffer as the input source, they need some way to feed fuzzing inputs into the network programs. There are three known ways now. AFL recommends to use Preeny (desock) [28], a hook-based tool, to simply redirect stdin to sockets hijacked by the tool [3], AFLNET [15] sends inputs through ordinary sockets to the network programs, and users could also modify the program source codes to make the programs read packets directly from memory buffers other than real network interfaces, like the Google OSS-Fuzz project does to openssl [10]. The first two ways may limit the execution speed of fuzzing (comparing with the desockmulti tool proposed in this paper). The third way may be difficult if the original developers of the programs do not expect such modification, and is also impossible for closed source programs. A very recent work AFLNET [15] proposed to combine coverage-based fuzzing with automated state model inferencing. While the fuzzing generates new message sequences to cover new states, the inferred state model guides how to do the fuzzing. AFLNET is shown to outperform Boofuzz [26] and AFL [3] in both code coverage and vulnerability discovery [15]. However, it requires users to write codes to extract partial information like the response codes from messages.

There are also some recent studies on the fuzzing in IoT domain. IoTFUZZER [38] is a new blackbox fuzzer which utilizes the mobile apps controlling IoT devices to do protocol fuzzing without protocol specifications. It indirectly mutates the protocol fields by mutating at data sources (e.g., string constants and inputs from system APIs). IoTFUZZER needs the mobile apps to fuzz network protocols, and is also limited to the fuzzing of functionalities related to the mobile apps. FIRM-AFL [39] uses AFL to fuzz IoT firmware, and uses augmented process emulation to fuzz programs at a higher speed. It mainly focuses on the fuzzing of ordinary programs in IoT firmware but not the network protocols. In [40], the authors propose a template-based fuzzing method to fuzz the MQTT protocol. The fuzzer is at the man-in-the-middle position between the client and the broker, and it selectively mutates the packets that match the specified types (e.g., PUBLISH messages). It provides templates to users to decide which fields to mutate, to alleviate the burden of writing codes like in Boofuzz [26]. However, users still need to know the specification (e.g., packet types) of the protocol. mqtt_fuzz [41] is an open-source tool designed for fuzzing the MQTT broker server. It could generate most of the MQTT packets for fuzzing. However, it has not been updated for five years, and does not support MQTT version 5.0 released in 2019 [21], not to mention the CoAP protocol that is also studied in this paper.

## 3. An Introduction to MQTT and CoAP

MQTT [21] and CoAP [22] are two important application-layer network protocols proposed in IoT [17,18,19,20]. MQTT is a publish/subscribe model protocol, and CoAP supports both the request/reply and publish/subscribe models [19,42,43]. The publish/subscribe model provides benefits that are crucial to the IoT network, like loose coupling and great scalability [19]. The two protocols (especially MQTT) are now widely used in the IoT network [44,45], and are also supported by IoT cloud providers like Amazon, Microsoft, and Google [24,25].

MQTT [21] is a publish/subscribe messaging transport protocol. It is light weight and has a simple design. It requires a small code footprint and limited network bandwidth. The protocol runs over TCP by default. Its clients could be publishers who publish application messages that other clients might be interested in, or subscribers who request application messages that they are interested in. Its server acts as an intermediary (broker) between clients which publish application messages and clients which have made subscriptions (please refer to Figure 1 for its architecture). The information delivered by MQTT is based on topics, and topics use topic level separator (i.e., “/”) to introduce structure into the topic name. When the subscribers send subscription, they use topic filters, which may contain wildcards (i.e., multi-level wildcard “#”, or single-level wildcard “+”) so they could subscribe to multiple topics. However, when publishers send publish messages, they could only use topic name (no wildcards included).

In a publish message of MQTT, it could indicate one of the three QoS levels: 0 for at most once delivery (messages are delivered using the best efforts and message loss can occur), 1 for at least once delivery (messages are assured to arrive but duplicates can occur), and 2 for exactly once delivery (messages are assured to arrive exactly once). An MQTT control packet consists of up to three parts [21]: Fixed Header that presents in all MQTT control packets, Variable Header and Payload that present in some MQTT control packets. In MQTT v5.0 there are 15 types of MQTT control packets in total (the AUTH type is newly added in v5.0). We list them in Table 2. We can see that most of these packets are in pairs (i.e., a CONTROL command and its ACK), except that for QoS 2, there are 3 ACKs for ensuring delivery exactly once.

CoAP [22] is a specialized web transfer protocol for constrained environments. It follows the REST (Representational State Transfer) architecture of the Web, but is optimized for machine-to-machine (M2M) applications. It is bound to UDP by default, but could be bound to TCP as well [46]. It can be logically considered as a two-layer protocol, a CoAP messaging layer used to deal with UDP and the asynchronous interactions, and a request/response layer for the REST-style methods and response codes. In the messaging layer CoAP defines four types of messages: Confirmable, Non-confirmable, Acknowledgement, Reset. For example, marking a message as Confirmable (CON) could provide reliability to the up-layer. In the request/response layer, CoAP defines and uses GET, PUT, POST, and DELETE methods like HTTP, and uses a token field to match responses to requests independently from the underlying messaging layer (which uses a Message ID field for the similar purpose). CoAP also defines a URI scheme like HTTP, with the prefix “coap://” (or other variants like “coap+tcp://” for the TCP transport layer case).

The CoAP protocol also supports options, and it uses an Observe option to make CoAP clients can “observe” resources in a publish/subscribe model [23]. The process could be as follows. A client sends an extended GET request (the Observe option is set to 0) to the server to register its interest in a resource. Whenever the state of the resource changes (e.g., by a PUT request from others), the server notifies each observing client by a response. In the response the token is the same as the token in the original GET request, and the Observe option is set to a sequence number for reordering detection.

The security of the MQTT and CoAP protocols are very important, since they may be deployed in hostile environments. Both of them can be secured by either TLS or DTLS (Datagram Transport Layer Security), depending on whether TCP or UDP is used as the transport layer protocol [21,22]. Mutual authentication between the client and the server can also be added [21]. Researchers also formally verified the protocols [47], studied the possible attacks [48], and proposed intrusion detection for them [49].

## 4. MultiFuzz

In this section, we give an overview of MultiFuzz first, and describe its techniques in detail in later subsections.

### 4.1. Overview

The basic idea of MultiFuzz is to make the fuzzer support multiple connections when fuzzing a single seed. Thus, it could simulate the processes of the IoT publish/subscribe protocols (as well as other multiparty protocols), since during fuzzing, each connection could represent any party in the protocols. We make necessary changes from the seed input to the execution of a PUT.

**Figure 2:**
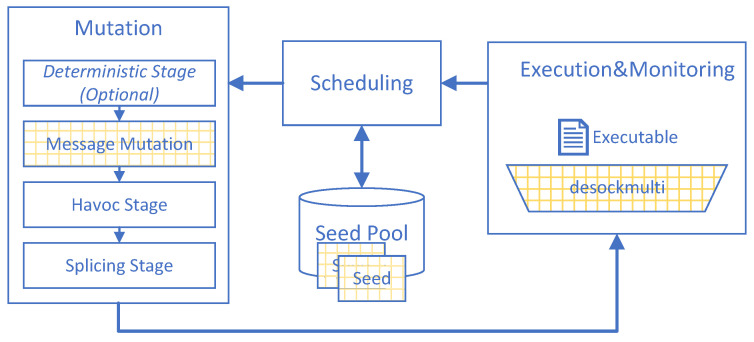
The architecture of MultiFuzz. It has the same architecture as other coverage-based fuzzers like AFL [3], with the changes highlighted by yellow grids.

We show the architecture of MultiFuzz in Figure 2. It is similar to other coverage-based fuzzers like AFL [3] (MultiFuzz is implemented based on AFL), and can be divided into four modules as well. The different parts are highlighted by yellow grids. First, the seed pool stores all initial seed inputs and newly found interesting inputs. MultiFuzz includes a new seed format to store the information of multiple connections in a seed. Second, the scheduling module selects seeds from the seed pool and sends to the mutation module. The scheduling module also later checks with the execution and monitoring module for any new inputs that have interesting paths, and saves such new inputs into the seed pool for future use. The scheduling module is unchanged in MultiFuzz. Third, the mutation module mutates the seed inputs and sends to the execution and monitoring module. The mutation module can be further divided into three stages in AFL [3]: the deterministic stage (using some predefined operations like “bitflip” and “arithmetic inc/dec”), the havoc stage (making stacked changes using previous operations), and the splicing stage (splicing the seed with another randomly selected seed). The deterministic stage takes a long time and can be skipped by using the “-d” option. MultiFuzz adds a new message mutation stage before the havoc stage to make message-aware mutation at a higher level. The execution and monitoring module executes the PUT and feeds inputs into it, and monitors the results like any crashes or other misbehavior. The module previously may use desock (Preeny) [28] to hook socket functions in order to feed inputs into the PUT, but MultiFuzz uses a faster and multi-connection-oriented tool desockmulti instead.

### 4.2. Augmenting Seeds with Multi-Connection Information

We need a new seed format for storing the information of multiple connections. Existing fuzzers [3,10,15] assume that only a single connection is made to the PUT when fuzzing with a seed, thus, they could directly store all raw messages in a seed, without any extra information. During fuzzing, the fuzzers start a connection (either really [15], by hooking [3,28], or virtually [10]), and send all the messages to the PUT through the connection. In MultiFuzz, however, we need to start multiple connections for a seed input, so which messages belong to each connection must be determined. We also want the determination to be definite but not random, because we want the fuzzer to be stable (i.e., running the PUT with the same seed multiple times exercising the same path). We once considered embedding the meta information (like the number of connections and the lengths of messages) in the file name of a seed, and storing the real messages in the seed file. However, we do not use the method because: (i) file names have limited lengths in some file systems (e.g., 255 bytes in ext4), which limit the number of messages in a seed, (ii) the file names may already have meanings, for example, AFL [3] embeds id, parent, and mutation operation in the seed file name, (iii) sometimes we may want to fuzz the meta information like the length of a message, then we need to design extra fuzz operations. We finally decide to embed the meta information into the seed content as well, and experiment results confirm that it works well (Section 5).

**Figure 3:**
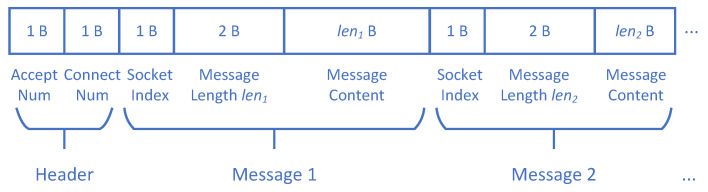
The new seed format.

**Figure 4:**
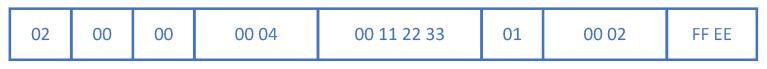
An example seed in the new format (hex encoding).

We design a new seed format for embedding multi-connection information and show it in Figure 3. We want to keep the seed compact so we use a binary form format. Each seed now contains a HEADER and one or more MESSAGEs. The HEADER occupies two bytes, and each represents an unsigned value ([0–255]). The first byte is the number of sockets that connect to the PUT’s accepting (i.e., listening) socket. The second byte is the number of sockets that the PUT connect to others (the connect num is not used yet since the servers we fuzzed do not initiate connections to others). In each MESSAGE, there is a 1-byte unsigned value ([0–255]) representing the index of the socket the message belongs to (i.e., which socket the message sent through), a two-byte unsigned value ([0–65535], little endian) representing the length of the message $len_{i}$, and $len_{i}$ bytes representing the message content. In a seed, different MESSAGEs could have the same socket index and all of them will be sent through the socket. We show a seed example in Figure 4. Its accept num is 2, so the fuzzer will initiate two sockets connecting to the PUT’s accepting socket. The fuzzer will send the 4-byte message “00 11 22 33” through the first socket (socket index = 0), and send the two-byte message “FF EE” through the second socket (socket index = 1).

### 4.3. The New Message Mutation Stage

Existing mutation stages are not message-aware and are inefficient in stimulating the state transitions of protocols. We would like to design a mutation algorithm that does not need users to do extra work, but still provides good results. Our basic idea is to make full mutation on the message sequences, and rely on the existing evolution mechanisms in coverage-based fuzzing to approach new protocol states. For example, suppose currently there are three messages A, B, and C as separated seeds, there are handleA, handleB, and handleC functions handling these kinds of messages respectively (a common design pattern), and the PUT enters $state_{A}$, $state_{B}$, and $state_{C}$ after handling messages A, B, and C respectively and only the transitions $state_{A}$->$state_{B}$, and $state_{B}$->$state_{C}$ are allowed. Then, if there is a bug that only occurs in $state_{C}$ (i.e., after processing A, B, and C), it would be nearly unlikely to generate seed A‖B‖C (“‖” represents concatenation) with existing mutation stages. However, with our message mutation algorithm proposed next in this subsection, it would quickly generate input A‖B based on existing seed A and seed B (and would be saved as a new seed since it has a new path), also quickly generate the wanted A‖B‖C input (either based on the new seed A‖B, or directly based on A, B, and C). We also need to consider the special mutation requirements for our multi-connection scenarios.

We design a new message mutation algorithm, which makes stacking changes like the existing havoc stage in AFL [3], and show it in Algorithm 1. We add the message mutation stage before each havoc stage of a seed. We add it here because the deterministic stage is optional and called only once for a seed, and the splicing stage also reuses the havoc stage to mutate inputs after each splicing. In the algorithm, we first decide the number of stacking changes with a constant MF_STACK_POW2 (the constant is set to 3 now, so the maximum number of stacking changes is 16). Then each time we randomly choose one operation out of the five possible ones. The first operation is to add or minus the numbers (i.e., accept num or connect num) in the header of the seed. The second operation is to add or remove a whole connection. Note that in the operation (and next operation) we may need messages for stuffing, and we get them from randomly chosen seeds. We do not limit to the initial seeds because new types of messages may be discovered during fuzzing. The third operation is a sequential change, which is to add or remove a message at the end of a connection. The fourth operation is like the third one, but is to add or remove a message at a random position in the connection. The fifth operation is to choose a random connection from the seed and switch the positions of two random messages in the connection.
**Algorithm 1** Message mutation algorithm**Input:** seed (its connection information already parsed) **Output:** output buffer
1:use_stacking = 1 << (1 + UR(MF_STACK_POW2))   // UR is random function2:**while** use_stacking > 0 **do**3:    Choose 1 out of below 5 operations randomly:4:      1. choose 1 out of the 4 operations randomly: to add/minus the accept num/connect num5:      2. choose to remove or to add a connection (if to add, further choose to add a single-message connection or to duplicate a full connection from a random seed)6:      3. add a random message to, or remove a message from the end of a random connection in the seed7:      4. like operation #3 but the change position is random in the connection8:      5. switch two randomly chosen messages of a connection in the seed9:    use_stacking = use_stacking - 1



### 4.4. desockmulti, A Fast and Multi-Connection-Oriented De-Socketing Tool

Since the seed input of MultiFuzz has a different format and is multi-connection-oriented, we need a new tool to feed the input into a PUT. The community usually uses the desock module of Preeny [28] to work with AFL [3] (recommended by AFL [3] for no code modifications needed). desock uses LD_PRELOAD to hook the socket(), bind(), listen(), and accept() functions. It uses two threads to synchronize a socketpair to stdin and stdout. However, its design makes it unable to accept multiple connections for a server (since all new sockets will be duplicated from the socketpair through dup() calls). In addition, the original purpose of Preeny is to interact with binaries locally; therefore, it is not optimized for fuzzing. For example, desock uses poll() to keep reading from stdin. Then, first it needs an extra thread to keep calling poll(). Second, it is unnecessary since in fuzzing the whole input is provided at a time and no poll() calls are needed. We find the performance of desock is indeed limited in fuzzing (Section 5.4).

We design and implement a new tool, desockmulti. It has the following advantages:

- desockmulti supports the new seed format.
- desockmulti can initiate multiple connections (i.e., one or more) to a PUT, and it could be a replacement of desock, which can only initiate one connection.
- desockmulti is optimized for fuzzing, and is 10x faster than desock.

**Figure 5:**
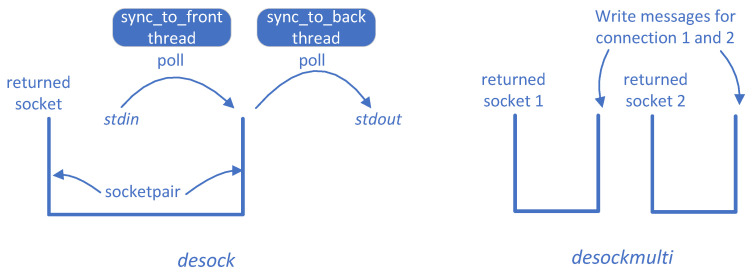
The design of desock and desockmulti.

We describe the design of desockmulti in detail. It also uses LD_PRELOAD for hooking the major socket functions socket(), bind(), listen(), and accept(), and uses UNIX sockets to simulate the original INET/INET6 sockets. However, the relationships of the sockets in desock and desockmulti are different, as shown in Figure 5. In desock, only a single socket pair created by the socketpair() system call is used. One socket of the pair is returned to the PUT (though the socket may be duplicated to other file descriptors by dup() in the hooked accept() function), and the other socket’s read stream and write stream are synchronized to stdout and from stdin respectively by two threads. In desockmulti, multiple socket pairs are created for multiple connections, and one socket of each pair is returned to the PUT. However, these socket pairs are not created by the socketpair() system call but by ordinary connect() and accept() calls. This is because multiple accept() calls are the only valid way to make the PUT process multiple new connections as usual. In addition, other hooked socket functions work in more “real” ways as well. For example, in the hooked bind(), we really bind the socket at an address.

We improve the performance of desockmulti mainly with the following optimizations: (i) we read all the content of a seed at a time without using poll(), since no interaction is needed during fuzzing, (ii) we removed the calls to dup2() which seems to be slow, (iii) we use the abstract socket address (https://www.man7.org/linux/man-pages/man7/unix.7.html) in Linux system to remove the socket’s relation with ordinary filesystem, (iv) and we remove the use of threads which is unnecessary in the new design, even we are using connect() and accept() to create socket pairs. We discovered some optimizations (e.g., (ii) and (iv)) through profiling (e.g., strace and detailed logging). Comparing with desock, desockmulti has more optimizations like (i), (ii), and (iv), which make it 10× faster in fuzzing (Section 5.4).

### 4.5. Other Implementation Details

We share some other implementation details here. We implement MultiFuzz based on AFL [3] and we add an option “–l” for enabling MultiFuzz. Since the seeds are mutated randomly and some numbers like the socket index may become out of their bounds (i.e., accept num + connect num), we use the numbers modulo their bounds, instead of treating the seeds as invalid. We add a method multifuzz_generate() for the message mutation of MultiFuzz, and call it before each of the stacking havoc mutation. We make desockmulti support the original seed format by an environment variable USE_RAW_FORMAT. When the variable is set desockmulti behaves like a faster desock. MultiFuzz uses messages as seed inputs like other coverage-based fuzzers. We use Wireshark (https://www.wireshark.org/) to capture the messages, and develop a Python script to dump messages from the captured pcap files.

## 5. Evaluation

### 5.1. Experiment Settings

In order to evaluate MultiFuzz, we selected two famous MQTT and CoAP protocol implementations, Eclipse Mosquitto [29] and libCoAP [30]. Eclipse Mosquitto [29] is a message broker that implements the MQTT protocol versions 5.0 [21], 3.1.1, and 3.1. It also provides mosquitto_pub and mosquitto_sub command line MQTT clients. We used its latest release version 1.6.10 for fuzzing. We built Mosquitto by not enabling TLS (make WITH_TLS=no) since we did not want to fuzz the TLS codes. When collecting fuzzing seeds, we used the mosquitto_sub and mosquitto_pub clients to connect to the local broker server, and sent a subscribe request and a publish request respectively. Then, we used Wireshark to capture the packets and used a custom Python script to dump messages as we mentioned before. We got 10 seeds for Mosquitto. libCoAP [30] is a C-Implementation of CoAP that provides core functionality for the development of resource-efficient CoAP servers and clients. It supports extensions like resource observation [23], TCP [46], block-wise transfer, FETCH/PATCH, and No-Response. It provided CoAP-client and CoAP-server and we used CoAP-server as the PUT. We used its latest release version 4.2.1 for fuzzing. We also built with TLS disabled. Since our desockmulti did not support UDP yet (desock does not support it either), we used TCP as the transport layer protocol for CoAP-client and CoAP-server. For collecting seeds, we used two CoAP-client instances to observe and put a resource respectively (realizing the resource observation). Following the same method, we got 31 seeds for libCoAP. We also built both projects without Address Sanitizer (ASan) [50] enabled for faster fuzzing speeds, and we later built them with ASan enabled for analyzing crashes and program paths (We did not consider the detection of data race or race condition in this paper, hence we did not use sanitizers like ThreadSanitizer, or specially design any mechanisms to boost concurrency).

Similar to AFL [3] (working with desock), MultiFuzz could be run with following command in the console: LD_PRELOAD=/path/to/desockmulti/desockmulti.so ./afl-fuzz -l 0 -d -i testcase_dir -o findings_dir —/path/to/program [...params...], where “-l” is for enabling MultiFuzz as mentioned before, and “0” is for ordinary-initial-seed case (“1” is for new-format-initial-seed case, i.e., format shown in Figure 3).

We chose AFL [3], and two state-of-the-art fuzzers MOPT [14] and AFLNET [15] to compare with MultiFuzz. AFL [3] is one of the most famous coverage-based fuzzers, and we use its latest version 2.52b. MOPT is a recently proposed fuzzer that uses Particle Swarm Optimization (PSO) algorithm to find the optimal probability distribution of mutation operators [14]. AFLNET is a more recently proposed fuzzer that is based on AFL as well, but uses automated state model inferencing to work with coverage-based fuzzing [15]. AFLNET needs users to write codes to extract response codes from messages, and we implement them as required. Basically, we used the higher 4 bits of the first byte of a MQTT message, and the second byte of a CoAP message, as the response code respectively [21,22]. We ran AFLNET with its default settings (i.e., -D 10000 -q 3 -s 3 -K -R). We skipped the deterministic stage during the mutation (which is the default configuration of AFLNET) for all the fuzzers (including the MOPT fuzzer, which automatically skips the deterministic stage after a while [14]). We used the desock module of Preeny [28] to work together with AFL and MOPT. We also used the same seeds set for all fuzzers.

All the experiments ran on a server configured with 2 Intel(R) Xeon(R) CPU E5-2640 v4 @ 2.40 GHz processors, 64 GB RAM, and with 64-bit Ubuntu 20.04 LTS. All the fuzzers ran 2 days with a single fuzzer instance, since it was recommended to run more than 24 h [31].

### 5.2. Path and Crash Discovery

**Figure 6:**
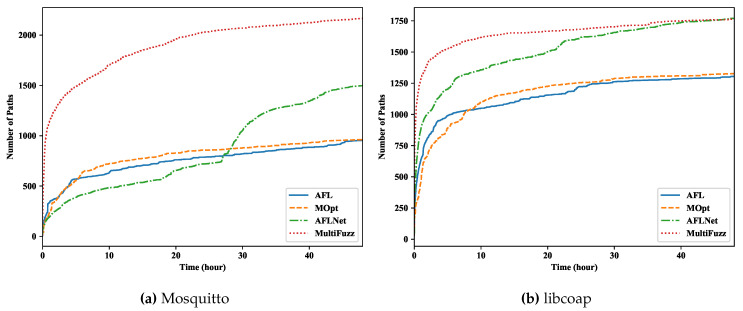
The paths discovered by different fuzzers.

The number of discovered program paths is one of the most important indicators of a fuzzer’s ability, and we show the experiment results in Figure 6 (we show the results of one run and different runs have similar results). We can see that all fuzzers had a similar trend: finding more paths quickly at the beginning and reaching a plateau later. An exception was AFLNET in fuzzing Mosquitto, which had a sudden growth in the middle (because of the big seeds produced at that time, and we will further analyze it later in this subsection). In both projects, we can see that MultiFuzz discovered paths much faster than other fuzzers at the beginning. When later most paths were already found, the discovery of MultiFuzz slowed down and AFLNET could catch up. However, it seemed difficult for AFL and MOPT to find the same paths even fuzzing for a long time. MultiFuzz eventually discovered 2166 paths when fuzzing Mosquitto, which was 44.6% more than AFLNET (1498), 126.6% more than AFL (956), and is 125.4% more than MOPT (961). In the case of libCoAP, the results were similar. MultiFuzz discovered 1763 paths, which was similar to AFLNET (1769), but was 35.2% more than AFL (1304), and was 32.9% more than MOPT (1327). We also checked the found paths (i.e., seeds/queue entries) and confirmed that MultiFuzz did find new message types that were not in the original seed set, e.g., the PUBCOMP [21] message. The results confirmed the hardness of fuzzing network protocols, since even though MOPT could optimize the selection of mutation operators, it could not discover many more paths than AFL. The AFLNET fuzzer indeed could discover more paths than AFL, which proves that it utilized the message response codes we returned in codes very well. Finally, as a fuzzer that did not need extra information or codes from users, MultiFuzz discovered many more paths than similar fuzzers AFL and MOPT.

**Figure 7:**
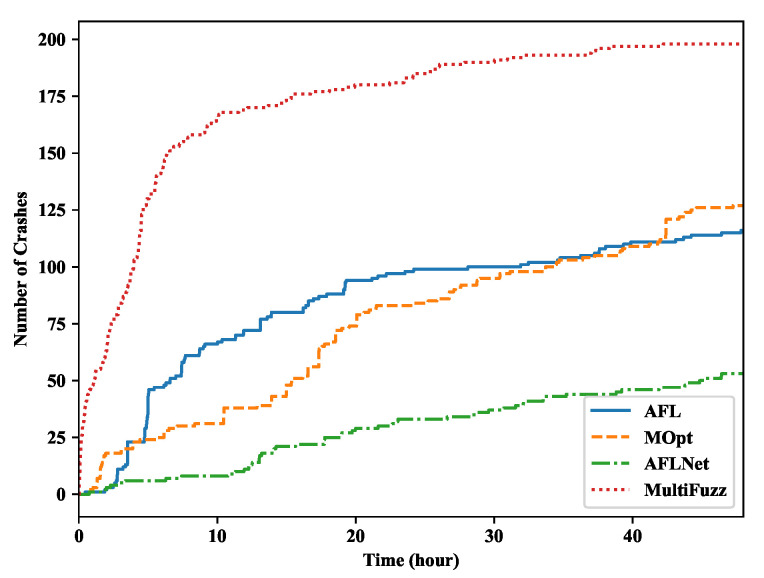
Crashes found in libcoap.

We compare the number of crashes found by different fuzzers in Figure 7. Since all the fuzzers could not find any crashes in Mosquitto, we only show the results of libCoAP. MultiFuzz also found crashes more quickly than other fuzzers. MultiFuzz eventually found 198 unique crashes, which was 70.7% more than AFL (116), 55.9% more than MOPT (127), and 273.6% more than AFLNET (53). The results indicated that MultiFuzz could search for bugs more quickly than the fuzzers that were not optimized for the fuzzing of network protocols. AFLNET did not perform well here, which may be due to it focusing more on higher level message mutations, but not on the detail program logics.

**Figure 8:**
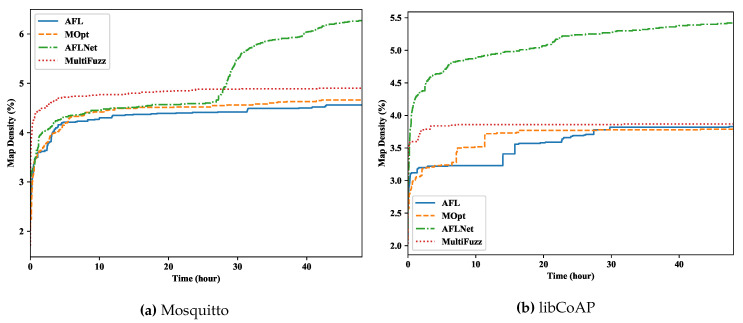
Bitmap density comparisons.

We used the bitmap density to check the code coverage of the fuzzers, and show the results in Figure 8. Coverage-based fuzzers usually use a fixed-size (e.g., 65536 bytes in AFL [3]) bitmap to store the coverage of all inputs. Each Control Flow Graph (CFG) edge of the PUT was mapped to a location in the bitmap (without considering collisions in high density cases [11]). Thus, the bitmap contained the accumulated edge coverage of all inputs. The bitmap density represented the ratio of locations in the bitmap that have values (i.e., edges travelled). We can see that MultiFuzz had a slightly higher bitmap density than AFL and MOPT, which was expected considering our message mutation algorithm.

However, AFLNET had a much higher bitmap density than other fuzzers, which shows that it could travel more edges. First, this may be due to its ability to intentionally exercise rarely exercised protocol states. Especially for libCoAP, we mainly generated publish/subscribe related message seeds, which may have left more room for the fuzzers to explore. Second, we further checked the queue entries (i.e., seeds) of AFLNET, and found their sizes were much bigger than other fuzzers (for Mosquitto, the sizes grew a lot only around the middle of the fuzzing process, but for libCoAP, the sizes grow quickly even at the beginning). Many seed sizes of AFLNET were several hundreds of KB (some were even over 1 MB), and thousands of messages were inside a single seed. For comparison, the seed sizes of MultiFuzz and other fuzzers were usually less than 1 KB and were tens of KB at most, and only several or tens of messages were inside a single seed. Bigger size seeds may make the execution slower (Section 5.4). They also make the execution unstable (i.e., having different paths for the same seed) as well. We found the stability ratio (i.e., 1—the ratio of variable bytes in the bitmap) of AFLNet dropped to about 50% in Mosquitto and about 20% in libCoAP (other fuzzers all were over 90%). However, on the other side, big size seeds may also have introduced dynamic message processing behaviors (e.g., different read/write orders), which seemed to be a plausible method to increase code coverage.

### 5.3. Effects of the Multi-Connection Design and the Message Mutation Algorithm

We know that the multi-connection design and the message mutation algorithm make the fuzzing of multiparty protocols sound and more efficient, however, it may be unclear that how much they improve the ability of MultiFuzz. We here make two variations of MultiFuzz: MultiFuzz (SingleConn., NoMsgMuta.) and MultiFuzz (NoMsgMuta.). In the first variation, we treat the seeds as in the original seed format that a seed only includes the messages of a single connection (i.e., by setting the USE_RAW_FORMAT environment variable when using desockmulti), and we disable the message mutation algorithm. In the second variation, we use the new seed format and desockmulti as normal, but disable the message mutation algorithm.

**Figure 9:**
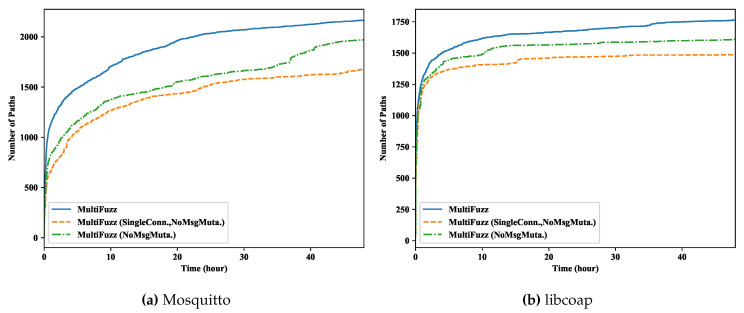
The paths discovered when disabling both the multi-connection design and the message mutation algorithm (MultiFuzz (SingleConn., NoMsgMuta.)), and disabling only the message mutation algorithm (MultiFuzz (NoMsgMuta.)).

We show the paths discovered by MultiFuzz and its variations in Figure 9. We can see that MultiFuzz constantly outperformed its two variations, and MultiFuzz (NoMsgMuta.) constantly outperformed MultiFuzz (SingleConn., NoMsgMuta.). For Mosquitto, MultiFuzz eventually discovered 9.9% more paths than MultiFuzz (NoMsgMuta.), and 29.2% more paths than MultiFuzz (SingleConn., NoMsgMuta.). For libCoAP, MultiFuzz discovered 9.6% more paths than MultiFuzz (NoMsgMuta.), and 18.6% more paths than MultiFuzz (SingleConn., NoMsgMuta.). We can clearly see that both the multi-connection design and the message mutation algorithm improved the path discovery ability of MultiFuzz, though they added some overhead at runtime.

**Figure 10:**
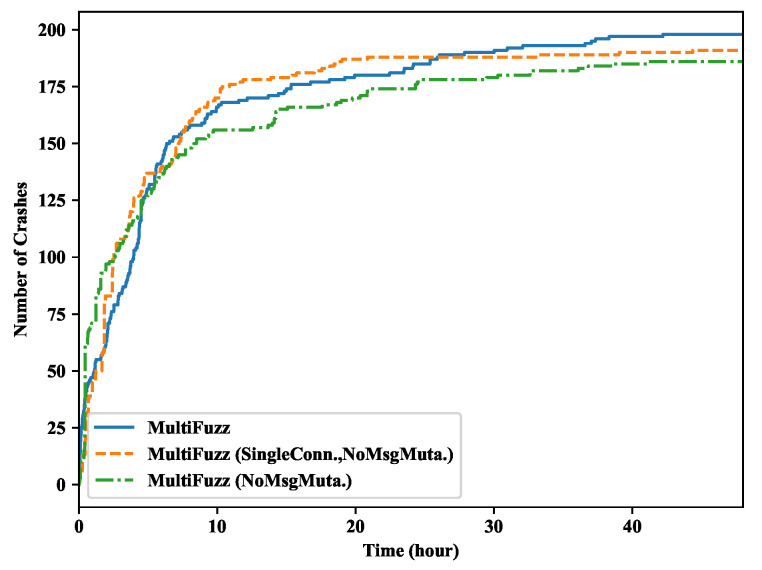
Crashes found in libCoAP by MultiFuzz and its variations.

We also show the number of unique crashes found in libCoAP by these three types of MultiFuzz in Figure 10. MultiFuzz only slightly outperformed its two variations, and it eventually discovered seven more crashes than MultiFuzz (SingleConn., NoMsgMuta.), and 12 more crashes than MultiFuzz (NoMsgMuta.). It seemed that the multi-connection design and the message mutation algorithm did not improve the crash-finding ability too much.

### 5.4. The Comparison of Execution Speeds

**Figure 11:**
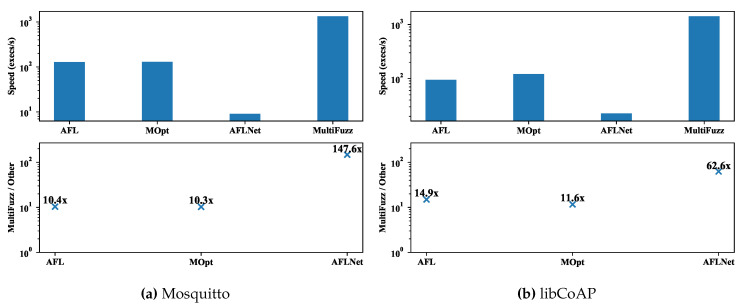
The execution speeds of different fuzzers, and the comparisons of MultiFuzz to other fuzzers.

We paid much attention to the performance of MultiFuzz (including the message mutation algorithm and the desockmulti module), and we show the execution speeds (executions/second) of different fuzzers, as well as the speed comparisons between MultiFuzz and others in Figure 11. We used tmpfs (https://www.kernel.org/doc/html/latest/filesystems/tmpfs.html) to store fuzzing outputs for faster execution, and we built the binaries for fuzzing without ASan enabled as we mentioned before. The average execution speed of MultiFuzz was 1333.9 execs/s for Mosquitto and 1412.4 execs/s for libCoAP. In contrast, the average execution speed of AFL was 128.8 execs/s and 94.6 execs/s respectively, which means MultiFuzz was 10.4× and 14.9× faster than AFL. MOPT was a bit faster than AFL, but MultiFuzz still was 10.3× and 11.6× faster than it. Since AFL and MOPT used the desock module of Preeny [28], most of the speed improvement of MultiFuzz was due the newly designed desockmulti tool. The execution speed of MultiFuzz (SingleConn., NoMsgMuta.) could be used as a quick reference as using AFL with desockmulti, since only a little extra initialization work was added in the variation. The average execution speed of MultiFuzz (SingleConn., NoMsgMuta.) was 1389.9 execs/s for Mosquitto and 983.8 execs/s for libCoAP, which is 10.8× and 10.4× faster than AFL with desock as well. The execution speed of AFLNET was slower than other fuzzers. It was only 9.0 execs/s for Mosquitto and 22.6 execs/s for libCoAP. We think it was mainly due to that AFLNET using real INET network socket to connect to a PUT. The INET network socket was known to be much slower than the UNIX socket. In addition, as we mentioned we found AFLNET generated much bigger size seeds (hundreds of KB) than other fuzzers (<1 KB), which also would slow down the execution speed.

### 5.5. Vulnerability Analysis

We manually investigated some of the found paths and crashes, and share the findings below.

**Memory leaks in Eclipse Mosquitto**. We found two memory leaks in Mosquitto, which could be repeatedly triggered by malformed requests and may cause Denial of Service (DoS) in the broker server. Both leaks were caused by the missing of free() calls during error processing. The first leak was in the handle__subscribe() method, at line 122 of src/handle_subscribe.c. When the broker processed a malformed MQTT v5.0 subscribe request, it called return MOSQ_ERR_PROTOCOL directly, without calling mosquitto__free(sub);mosquitto__free(payload); as in other error processing cases. The second leak was in the handle__publish() method, at line 112 of src/handle_publish.c. When it processed a malformed MQTT v5.0 publish request, it called return rc directly, without calling mosquitto__free(topic). We reported the leaks to the Eclipse Mosquitto project and the developers fixed them instantly (https://github.com/eclipse/mosquitto/commit/94d04136f8beedbd34ca16158b5be3111a9cb7b4). The fixes should be available in the next version 1.6.11.

**assert()****failures and a memory leak in libCoAP**. We found that most of the crashes were due to the failures of assert() calls, e.g., the failure of assert(pdu->max_size > 0) in the coap_write_block_opt() function, at line 77 of src/block.c. Such failures usually did not have impacts on production builds technically, since they should be built with the NDEBUG preprocessor macro defined, and the assert() method did nothing then. However, they could be reminders to the developers that unexpected things happen. For example, the memory leak we describe next has an assertion failure too. The memory leak was in the handle_request() method, at line 2208 of src/net.c. When the CoAP-server processed a malformed request in the handle_request() function, the call to the coap_add_token() method failed. However, only coap_log(LOG_WARNING, "cannot generate response\r\n") was called in the case, without a coap_delete_pdu(response) call to free the PDU timely. The memory leak could also be repeatedly triggered by malformed requests so it could cause DoS as well. We reported the leak to the libCoAP project and was acknowledged as well (https://github.com/obgm/libcoap/issues/535).

## 6. Conclusions

This paper presented a coverage-based fuzzer MultiFuzz, which initiates multiple connections to a program under test, to soundly support the fuzzing of multiparty protocols like the publish/subscribe protocols in IoT. MultiFuzz contains a new seed format, a message mutation algorithm, and a new de-socketing module desockmulti. We used MultiFuzz to fuzz the Eclipse Mosquitto project and libCoAP project, and reported our found vulnerabilities to the developers (all were acknowledged and fixed). We also showed that MultiFuzz found more paths and crashes, comparing with AFL, and two state-of-the-art fuzzers, MOPT and AFLNET. We think MultiFuzz is not limited to the fuzzing of IoT publish/subscribe protocols, and could be used to soundly fuzz other multiparty protocols as well. In addition, we believe the desockmulti module of MultiFuzz could benefit the community after open-sourcing, since it is similar to the widely used tool desock (Preeny) but is 10x faster.

## Figures and Tables

**Table 1:** Fuzzer comparisons. “Partial” means some knowledge or work is needed.

Fuzzer	Need Spec.	Need Coding	Support Multiparty	Message-aware	Taxonomy
Boofuzz (Sulley) [26]	Yes	Yes	**Yes**	**Yes**	blackbox
AFL [3]	**No**	**No**	No	No	greybox
MOPT [14]	**No**	**No**	No	No	greybox
AFLNET [15]	Partial	Partial	No	**Yes**	greybox
MultiFuzz (this paper)	**No**	**No**	**Yes**	**Yes**	greybox

**Table 2:** MQTT control packet types [21].

Name	Direction of Flow	Description
CONNECT	Client to server	Connection request
CONNACK	Server to client	Connect acknowledgment
PUBLISH	Client to server or server to client	Publish message
PUBACK	Client to server or server to client	Publish acknowledgment (QoS 1)
PUBREC	Client to server or server to client	Publish received (QoS 2 delivery part 1)
PUBREL	Client to server or server to client	Publish release (QoS 2 delivery part 2)
PUBCOMP	Client to server or server to client	Publish complete (QoS 2 delivery part 3)
SUBSCRIBE	Client to server	Subscribe request
SUBACK	Server to client	Subscribe acknowledgment
UNSUBSCRIBE	Client to server	Unsubscribe request
UNSUBACK	Server to client	Unsubscribe acknowledgment
PINGREQ	Client to server	PING request
PINGRESP	Server to client	PING response
DISCONNECT	Client to server or server to client	Disconnect notification
AUTH	Client to server or server to client	Authentication exchange
